# Modeling HIV-1 infection and NeuroHIV in hiPSCs-derived cerebral organoid cultures

**DOI:** 10.1007/s13365-024-01204-z

**Published:** 2024-04-10

**Authors:** Martina Donadoni, Senem Cakir, Anna Bellizzi, Michael Swingler, Ilker K. Sariyer

**Affiliations:** Department of Microbiology, Immunology and Inflammation, Center for Neurovirology and Gene Editing, Temple University Lewis Katz School of Medicine, Philadelphia, PA USA

**Keywords:** hiPSCs, Cerebral organoids, HIV-1, NeuroHIV, cART, Microglia, Astrocytes

## Abstract

The human immunodeficiency virus (HIV) epidemic is an ongoing global health problem affecting 38 million people worldwide with nearly 1.6 million new infections every year. Despite the advent of combined antiretroviral therapy (cART), a large percentage of people with HIV (PWH) still develop neurological deficits, grouped into the term of HIV-associated neurocognitive disorders (HAND). Investigating the neuropathology of HIV is important for understanding mechanisms associated with cognitive impairment seen in PWH. The major obstacle for studying neuroHIV is the lack of suitable in vitro human culture models that could shed light into the HIV-CNS interactions. Recent advances in induced pluripotent stem cell (iPSC) culture and 3D brain organoid systems have allowed the generation of 2D and 3D culture methods that possess a potential to serve as a model of neurotropic viral diseases, including HIV. In this study, we first generated and characterized several hiPSC lines from healthy human donor skin fibroblast cells. hiPSCs were then used for the generation of microglia-containing human cerebral organoids (hCOs). Once fully characterized, hCOs were infected with HIV-1 in the presence and absence of cART regimens and viral infection was studied by cellular, molecular/biochemical, and virological assays. Our results revealed that hCOs were productively infected with HIV-1 as evident by viral p24-ELISA in culture media, RT-qPCR and RNAscope analysis of viral RNA, as well as ddPCR analysis of proviral HIV-1 in genomic DNA samples. More interestingly, replication and gene expression of HIV-1 were also greatly suppressed by cART in hCOs as early as 7 days post-infections. Our results suggest that hCOs derived from hiPSCs support HIV-1 replication and gene expression and may serve as a unique platform to better understand neuropathology of HIV infection in the brain.

## Introduction

Human immunodeficiency virus (HIV) is a retrovirus isolated in 1983 and is the causative agent of acquired immunodeficiency syndrome (AIDS) (Barré-Sinoussi et al. 1983). Entry of HIV into target cells occurs through a multistep process that involves four different steps: attachment, co-receptor binding, fusion and entry (Wilen et al. 2012). Attachment is mediated by viral glycoprotein gp120 and CD4 cell receptor on the surface of the cells and is followed by co-receptor binding. These co-receptors are specific chemokine receptors: the CC-chemokine receptor 5 (CCR5), used by HIV R5 strains, and CXC-chemokine receptor 4 (CXCR4), used by HIV X4 strains (Berkowitz et al. 1998). Following attachment, the viral envelope protein fuses with the host cell membrane and the viral core containing the HIV RNA, viral proteins and enzymes can finally enter the host cell (Berger et al. 1999). After the initial infection, the virus can cross the blood-brain barrier (BBB), gaining access to the central nervous system (CNS), where it can establish viral reservoirs, eventually leading to neuropathogenesis (Zayyad and Spudich 2015). This can happen as early as 3 to 5 days after initial infection (Koenig et al. 1986; Whitney et al. 2014). The major cellular reservoirs in the brain are microglia and perivascular macrophages. Several studies have provided evidence of susceptibility and productive infection of HIV in these cells (Watkins et al. 1990; Peudenier et al. 1991; Ioannidis et al. 1995; McCarthy et al. 1998; Albright et al. 2000; Joseph et al. 2015), whereas HIV infection in astrocytes remains a controversial topic. Studies have shown HIV infection in astrocytes with expression of early viral proteins, but markers of HIV replication were not detected in these cells, leading to the belief that HIV infection in astrocytes is not productive (Brack-Werner 1999). Astrocytes are the most abundant cells in the CNS and are considered HIV reservoirs, playing an important role in HIV induced neuropathogenesis (Valdebenito et al. 2021; Wahl and Al-Harthi 2023). Neuronal cells are not infected by HIV, but viral proteins and neurotoxicity from glial activation may cause neuronal damage, leading to neuronal dysfunction and cell death (Kovalevich and Langford 2012). Furthermore, viral proteins such as Tat, Vpr and gp120 can contribute to neuronal dysfunction, either directly or indirectly through microglia stimulation resulting in the release of proinflammatory cytokines such as IL-1β and TNF-α (Gelbard et al. 1993; Yeung et al. 1995; Nicolini et al. 2008). Another consequence of HIV invasion in the brain is the induction of an inflammatory response, which may also contribute to the development of HIV-associated neurocognitive disorders (HAND) (Navia et al. 1986; Brown 2015; Hong and Banks 2015; Saylor et al. 2016). HAND includes a range of neurocognitive impairments, with a classification based on severity: asymptomatic neurocognitive impairment (ANI), mild neurocognitive disorder (MND), and HIV associated dementia (HAD). Diagnosis of HAND involves neuropsychological testing and assessments of functional status (Antinori et al. 2007). Severity of HAND has changed since the beginning of the HIV/AIDS epidemic. In the beginning, HAD, the most severe form of HAND, was diagnosed in 20–30% of HIV patients (González-Scarano and Martín-García 2005). With the start of combination antiretroviral therapy (cART), HAD frequency has significantly decreased, although 50% of people with HIV (PWH) present neurological disorders (Clifford and Ances 2013; Saylor et al. 2016). This percentage did not change in the post-cART era, but now the majority of HAND cases are diagnosed as MND or ANI (McArthur et al. 1993; Heaton et al. 2010). The introduction of cART also had an impact on the neuropathology in HAND. In the pre-cART era, neuronal loss and HIV encephalitis were considered to have major roles in HIV neuropathogenesis. However, with the advent of cART, these dysfunctions became less common, and they are no longer considered enough to account for neurological dysfunction. Consequently, the lack of clear neuropathological changes strictly related to HIV infection in patients receiving cART indicates that the underlying mechanism of HAND is more likely related to functional changes in neurons (Gelman 2015; Saylor et al. 2016).

Gaining insight into the neuropathology of HIV is crucial for comprehending the mechanisms underlying cognitive impairment in PWH. Research on HIV neuropathology in humans has primarily relied on the examination and analysis of brain tissues obtained post-mortem. A significant obstacle in investigating HIV neuropathogenesis is the scarcity of suitable in vitro culture models that can recapitulate HAND pathology, considering that multiple types of cells within the CNS may contribute to the pathology. HIV infection cellular models in the CNS usually include cell lines and primary cells. Studies using these 2D culture models have enabled the identification and characterization of cellular processes associated with neuronal toxicity. In particular, viral proteins such as gp120, Tat, Nef and Vpr have shown to be toxic when exposed to neuronal cultures (Brenneman et al. 1988; Adamson et al. 1996; Piller et al. 1998; New et al. 1998; Kaul and Lipton 1999; Kaul et al. 2001; Chen et al. 2005; Mattson et al. 2005; Agrawal et al. 2007; Shah et al. 2013; Sami Saribas et al. 2017; Fields et al. 2017; Dong et al. 2019). To capture the nature of cell-to-cell interactions, it is necessary to employ a primary culture system that combines neurons and glial cells, resembling the composition of cells typically found in the intact brain. Various systems have been developed to investigate the impact of soluble factors released by microglia. Majority of the studies on neuroHIV were performed using immortalized microglial cell lines, macrophages derived from peripheral blood monocytes or primary human microglia that were isolated from tissues (Garcia-Mesa et al. 2017; Rawat and Spector 2017; Rai et al. 2020). Furthermore, the use of animal models, such as nonhuman primates (NHPs) and genetically modified rodent models, has played a significant role in advancing our knowledge of specific aspects related to HIV pathology. While these animal models have provided valuable insights, limitations remain in comprehending CNS infection in humans (Mallard and Williams 2018). Research on HIV neuropathology in humans has been constrained to the collection and examination of brain tissues post-mortem (Wiley et al. 1986; Koenig et al. 1986; Everall et al. 1991; Masliah et al. 2000). Thus, it is important to develop a 3D model of human origins to investigate HIV neuropathology.

Human induced pluripotent stem cells (hiPSCs) allow for easy generation of primary human neural cell types in culture through differentiation. As a result, they have been used to generate complex 3D cell systems, such as brain organoids, which contain multiple cell types. iPSC derived 3D-brain organoids were first generated by Lancaster et al. (Lancaster et al. 2013), which they named “cerebral organoids” (COs). These COs displayed functional neurons and glial cell populations, discrete brain regions, and proper dorsal cortical organization. The establishment of the CO model was a major breakthrough, allowing the human brain to be modeled in vitro with proper organization and cellular connections. The human CO model can be especially helpful in studying human neurotropic viral infections, such as HIV, which has been difficult to study due to limitations of the previously used 2D in vitro and in vivo models. In vitro models used lack a multicellular composition, which is crucial for studying HIV effects as HIV infects microglial cells, which in turn possess neurotoxic effects on neurons that are unable to be infected by HIV (Kovalevich and Langford 2012).

Here, we first developed and characterized hiPSCs from human dermal fibroblasts obtained from healthy individuals. We then developed and characterized a 3D model of human Cerebral Organoids (hCOs) containing the major cell types present in the CNS, including astrocytes, neurons, oligodendrocytes, and microglia. The hCOs are further characterized for the expression of HIV receptor and co-receptors. Finally, we demonstrated the susceptibility of hCOs to HIV infection, in the absence or presence of cART regimens by a series of biochemical, histological, and virological studies. We were able for the first time to show the efficacy of cART treatment in suppressing HIV replication in a human 3D CO model. The findings from our study provide a unique platform to enhance our understanding of the neuropathological aspects of HIV infection in the brain.

## Results

### Generation and characterization of hiPSCs

**Fig. 1:**
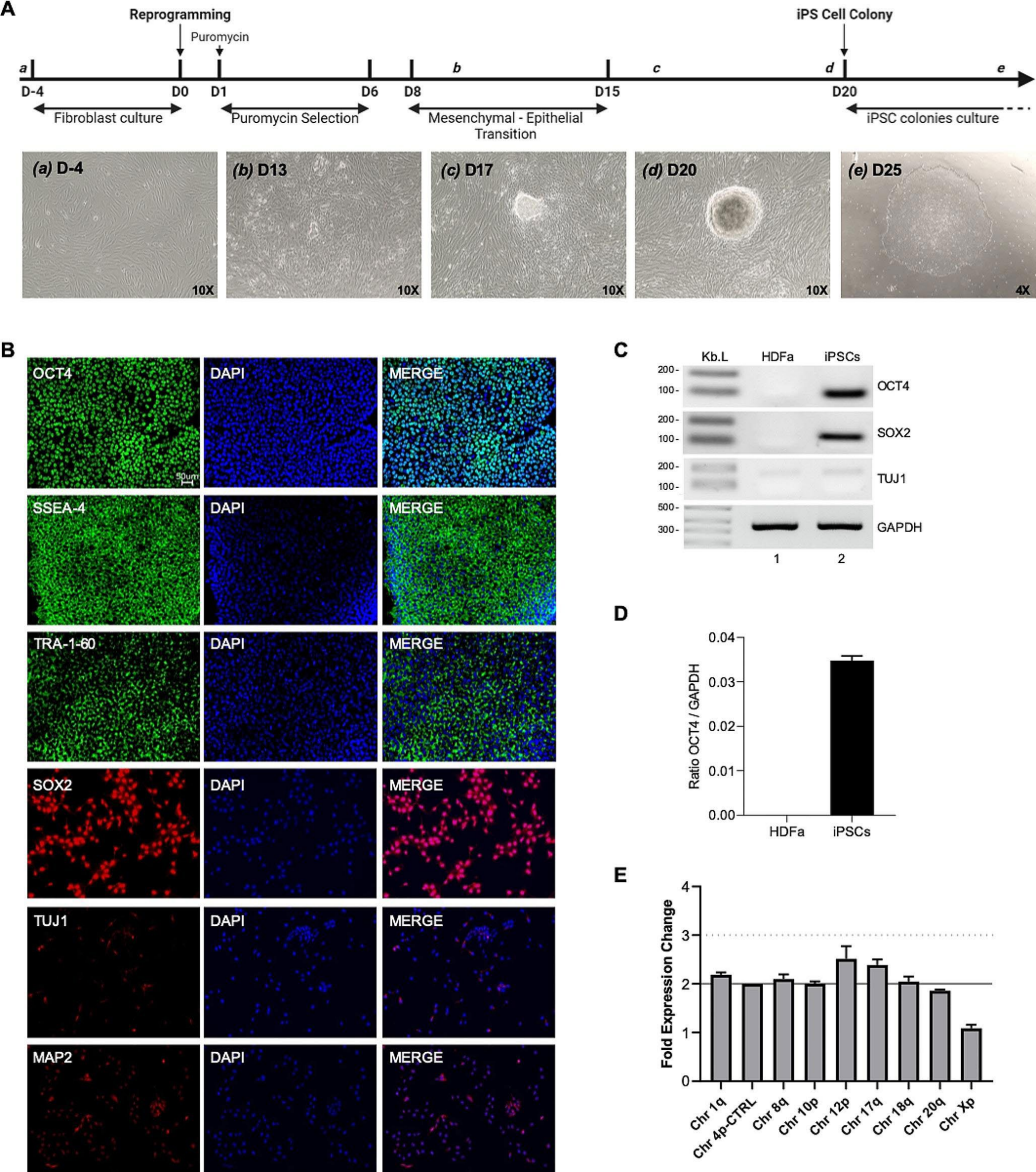
Generation and characterization of human induced pluripotent stem cells (hiPSCs) from human dermal fibroblast, adult (HDFa). **A**: Schematic timeline of generation of hiPSCs from HDFa. Representative images of reprogramming of HDFa to generate hiPSC single-cell colony cultures. **B**: Immunohistochemistry of hiPSCs for pluripotency markers, OCT4, SSEA-4, TRA-1-60 and SOX2, and neuronal differentiation markers, TUJ1 and MAP2. **C.** RT-PCR to confirm mRNA expression of pluripotency markers, OCT4 and SOX2, and neuronal marker TUJ1. GAPDH was also amplified from same samples as control. Line 1 is HDFa. Line 2 is hiPSC. **D.** hiPSC Genetic Analysis was performed for any possible karyotypic abnormalities reported for human iPSCs lines

Human primary adult dermal fibroblasts (HDFa) were reprogrammed to hiPSCs using a non-integrating, self-replicating RNA-based reprogramming vector that expresses Oct-3/4, Klf-4, Sox2, Glis1, and c-Myc transcription factors. Following reprogramming transfections, fibroblasts were selected using puromycin and kept in culture until the formation of iPSC colonies. Colonies were picked and expanded to form hiPSC cultures. As shown in Fig. 1A, robust morphological changes were observed during reprogramming and differentiation through the mesenchymal and epithelial transition, and clusters of pluripotent cells started emerging at day 17. At day 20, hiPSC colonies were large enough to be isolated and propagated. hiPSCs derived from HDFa were examined for the presence of pluripotency markers and neural differentiation markers. hiPSCs were stained positive for OCT4, SSEA-4, TRA-1-60 and SOX2 (Fig. 1B), that are considered the markers of undifferentiated cells. As expected, very few hiPSCs were slightly positive stained for neural differentiation markers, TUJ1 and MAP2 (Fig. 1B). These data were confirmed through RT-PCR and RT-qPCR (Fig. 1C and D). Total RNA was extracted from HDFa and iPSCs and were subject to analysis as described in “Material and Methods” section. As expected, mRNA expression of pluripotency markers OCT4 and SOX2 was not found in HDFa, but their expressions were detected in hiPSCs (Fig. 1C). Further, neural differentiation marker TUJ1 mRNA was not detected in either HDFa or iPSCs. Genomic DNA (gDNA) extracted from hiPSCs was also analyzed for genetical abnormalities, and no significant deletion or amplification were found in the chromosomes analyzed (Fig. 1E).

### Development of human cerebral organoids (hCOs) derived from hiPSCs

**Fig. 2:**
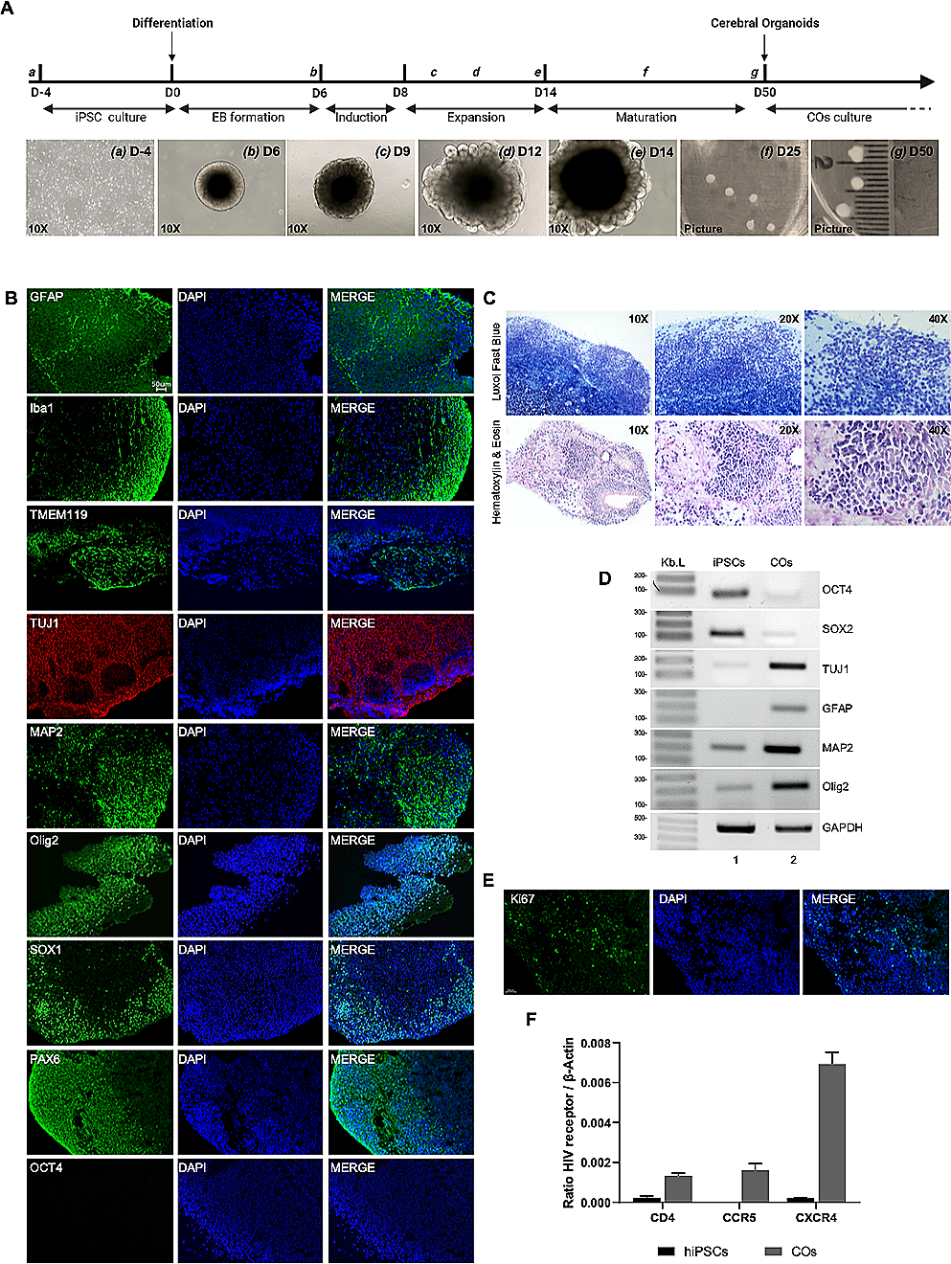
Generation and characterization of human cerebral organoids (hCOs) from hiPSCs. **A**: Schematic timeline of generation of hCOs from hiPSCs. Representative images of generation of hCOs showing Embryoid bodies (EB) formation (b), induction (c), expansion (d) and maturation steps (e-f). Fully mature hCOs representative image at day 50 (g) **B**: Immunohistochemistry (IHC) of hCOs for astrocytic structural marker, GFAP, microglial marker, Iba-1 and TMEM119, neuronal differentiation markers, TUJ1 and MAP2, oligodendroglia marker, Olig2, neural progenitor markers SOX1 and PAX6, and pluripotency marker, OCT4. **C.** Luxol Fast Blue for myelin staining and hematoxylin and eosin staining of mature hCOs. **D.** RT-PCR for mRNA expression of pluripotency markers, OCT4 and SOX2, neuronal markers, TUJ1 and MAP2, astrocytic structural marker, GFAP, and oligodendroglia marker, Olig2. GAPDH was also amplified from same samples. Line 1 is hiPSCs. Line 2 is hCOs. **E.** Immunohistochemistry (IHC) of hCOs for proliferation marker Ki67. **F.** RT-qPCR for mRNA expression of HIV-1 receptor, CD4, and co-receptors, CCR5 and CXCR4, in hiPSCs and hCOs. Three different lines of hiPSCs and three different hCOs were used. (Shown as mean ± SEM).

hiPSCs generated from HDFa were then differentiated into hCOs following an unguided protocol. This protocol allows the organoids into a self-directed organization into forebrain, midbrain, and hindbrain regions leading to the recapitulation of the entire brain (Lancaster et al. 2013; Qian et al. 2016; Swingler et al. 2023). Figure 2A highlights the hCOs generation timeline and images of hiPSCs differentiation into the hCOs. hiPSCs were first differentiated into embryoid bodies (EBs), with a diameter of 400–600 μm with smooth and round edges. EBs were then induced, and expansion was started with embedding in a matrigel. EBs started forming neuroepithelia, visible from budding from the surface and growing in size (Fig. 2A) at day 12 and 14. Finally, the maturation stage of organoids was initiated on day 14. After day 50, hCOs were considered mature and showed dense cores and translucent edges. hCOs generated in our study naturally formed four different fully differentiated cell types: astrocytes, neuronal cells, microglia and oligodendrocytes. hCOs were positively stained for GFAP (astrocytes), Iba1 and TMEM119 (microglia), TUJ1 and MAP2 (neuronal cells), and Olig2 (oligodendrocytes) (Fig. 2B). As expected, hCOs were negative for stem cell marker OCT4 expression but they were positive for neural stem cells markers SOX1 and PAX6. To further characterize hCOs composition, Luxol Fast blue staining was performed to show staining of myelin/myelinated axons, as well as hematoxylin and eosin (H&E) staining, to provide a comprehensive image of microanatomy of hCOs (Fig. 2C). Moreover, total RNA was extracted from hCOs to further analyze the expression of cell type markers through mRNA expression. RT-PCR analysis on hiPSCs (Fig. 2D, line 1) and hCOs (Fig. 2D, line 2) confirmed the expression of mRNA transcripts for OCT4 and SOX2 in hiPSCs while TUJ1 and GFAP were only present in hCOs. Interestingly, MAP2 and Olig2 mRNA transcripts were present in both hiPSCs and hCOs, but with a more robust expression in hCOs. (Fig. 2D). Cell proliferation within the hCOs were also assessed by Ki67 staining, a cellular marker specific for proliferating cells. As expected, a pool of cells was positive for Ki67, suggesting the presence of dividing cells (Fig. 2E). We then examined the expression of receptor and co-receptors involved in HIV-1 entry in RNA samples obtained from hiPSCs and hCOs. Interestingly, expression of CD4, CCR5 and CXCR4 were limited in hiPSCs (Fig. 2F). On the other hand, compared to the hiPSCs, expression of CD4, CCR5 and CXCR4 were all upregulated in hCOs. More interestingly, hCOs showed more than three-fold higher expression of CXCR4 than CD4 and CCR5.

### HIV-1 infection in hCO cultures

**Fig. 3:**
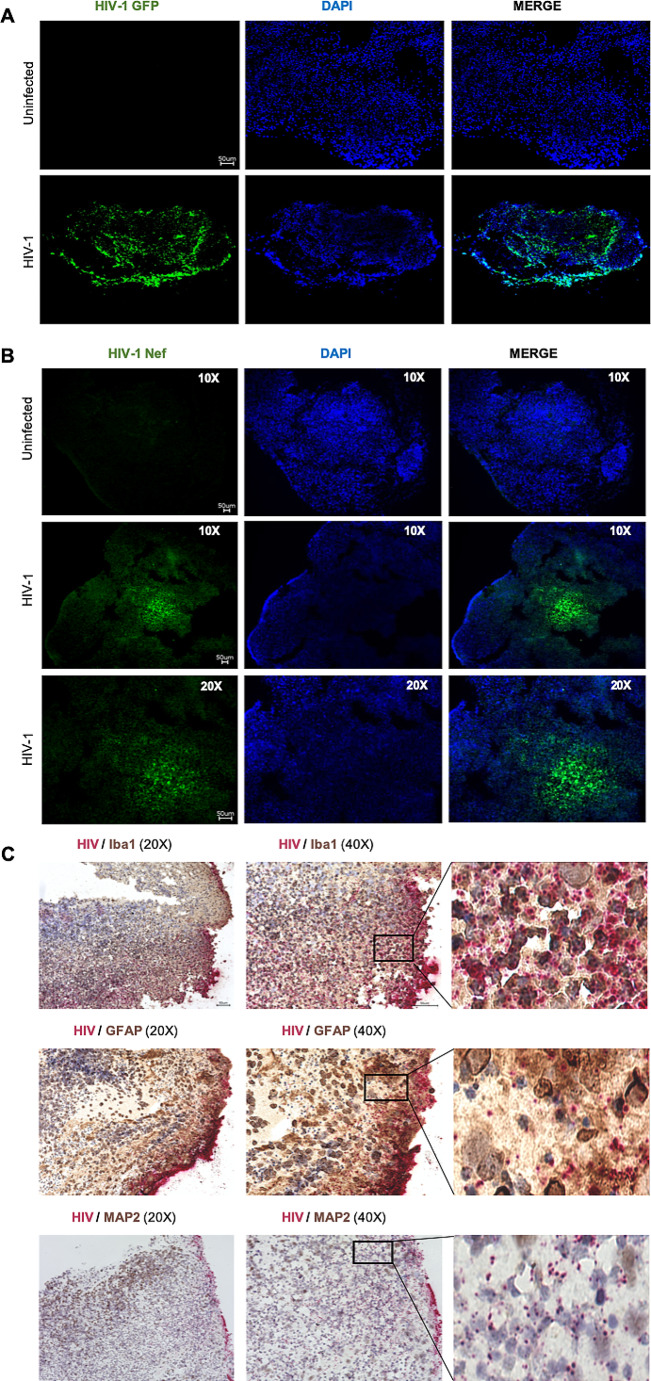
HIV-1 infection characterization in human cerebral organoids (hCOs). hCOs were infected with 300 ng of HIV-1 Bal-GFP virus. 4 days post-infection, hCOs were collected and fixed in 4% PFA for 16 h, after which they were cryo-embedded and prepared for staining. **(A)** Representative images of hCOs stained for GFP (green) and DAPI (blue). Images were taken at 10X magnification. **(B)** Representative images of hCOs stained for HIV-1 Nef (green) and DAPI (blue). Images were taken at 10X and details at 20X magnification. **(C)** Dual IHC-RNA Scope. Representative images of co-staining of HIV RNA (red) and cell type specific markers, Iba1, GFAP and MAP2 (brown). Images were taken at 20X and 40X magnifications. *N* = 3 hCOs.

To determine susceptibility of hCOs to HIV-1 infection, we infected hCOs with HIV-1 EGFP BaL reporter virus, as described in “Material and Methods” section. At 4 days post infections (dpi), hCOs were processed by immunohistochemical analysis for viral and cellular gene expressions. hCOs were first stained for GFP protein as indicative of viral infection. As shown in Fig. 3A, the GFP signal was widely spread across the entire organoids suggesting that hCOs are highly susceptible for HIV-1 infection. As expected, control/uninfected hCOs did not show immunoreactivity to GFP protein. To further investigate HIV-1 gene expression, hCOs were also stained for Nef, one of the HIV-1 accessory proteins. Interestingly, Nef was detected in clusters in the center and on the edge of the organoid sections (Fig. 3B). To gain more insight in the cell types infected with HIV-1, we utilized a dual technique which combined In Situ Hybridization– RNA scope with immunohistochemistry (IHC). hCOs infected with HIV-1 were first subject to RNA scope for the detection and visualization of HIV-1 RNA using a specific probe. hCOs were then processed with IHC for cell type markers, including Iba1 (microglia), GFAP (astrocytes) and MAP2 (neurons). As expected, a strong co-localization of viral RNA (red) and cellular Iba-1 (brown) were observed in microglial cells as co-stained in dark brown (Fig. 3C). In addition, while co-localization of HIV RNA and astrocyte marker GFAP was detected in some astrocytes, viral RNA had a limited co-staining in MAP2 positive neuronal cells.

### Suppression of HIV-1 infection by cART in hCOs

**Fig. 4:**
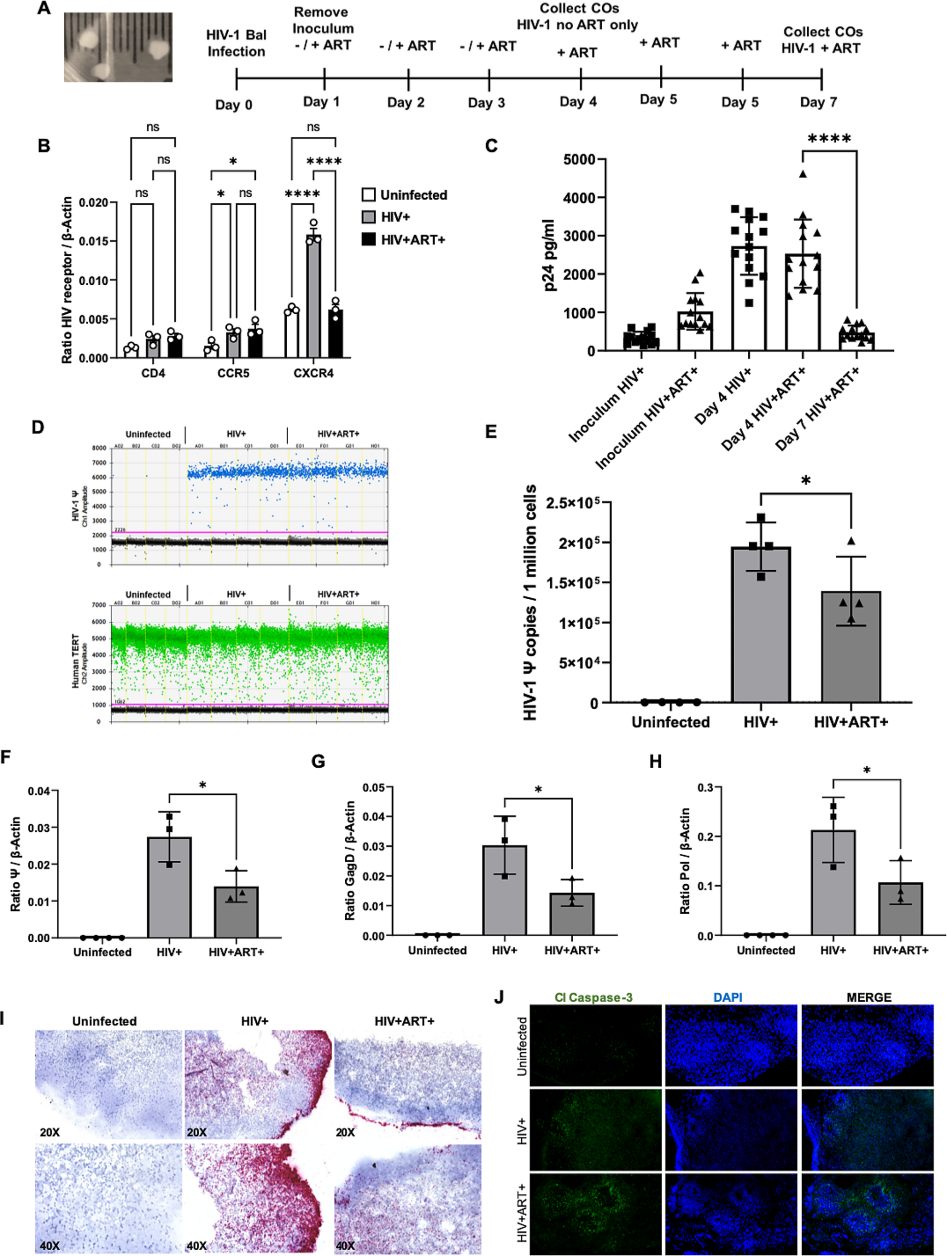
Characterization of HIV-1 infection in hCOs in the presence and absence of cART regimens. **A**: Schematic representation of experimental outline. hCOs were infected with HIV-1 Bal-GFP virus and treated with cART (Raltegravir, Tenofovir disoproxil fumarate, Emtricitabine) as outlined. **B.** RT-qPCR for mRNA expression of HIV-1 receptor, CD4, and co-receptors, CCR5 and CXCR4, in uninfected, HIV+, HIV + ART + hCOs. Three different hCOs per condition were used. **C**: Gag p24 ELISA on supernatant from hCOs uninfected, HIV + hCOs and HIV + ART + hCOs at day 1 (inoculum), day 4 and day 7 post-infections. Fourteen different hCOs per condition were used. **D-E**: gDNA from uninfected, HIV + and HIV + ART + hCOs was extracted and processed for ddPCR to detect Ψ and Human TERT (D) and data were shown as bar graphs per one million of cells (E). Four different hCOs per condition were used. **F-H**: RNA from uninfected, HIV + and HIV + ART + hCOs was extracted and processed for RT-qPCR for Ψ (F), GagD (G) and Pol (H) genes. Data were shown as bar graphs normalized to β-Actin. Three different hCOs per condition were used. **I**: hCOs were collected and fixed in 4% PFA for 16 h, after which they were cryo-embedded and processed for RNAScope to detect HIV-1 RNA (red). Three different hCOs per condition were used. **J.** hCOs were collected and fixed in 4% PFA for 16 h, after which they were cryo-embedded and processed for IHC for cleaved caspase-3. Three different hCOs per condition were used. (ELISA *n* = 14, RNA *n* = 3, gDNA *n* = 4. Shown as mean ± SEM, * *p* < 0.05, *** *p* < 0.001)

Our infection studies suggested that hCOs are highly susceptible for HIV-1 infection. We next investigated whether hCOs might be also a model for suppression of HIV-1 replication using cART regimens and form latent viral reservoirs to be utilized for neuroHIV studies. hCOs were infected with HIV-1 and treated with a cART regimen as schematized in Fig. 4A. All the hCOs with HIV-1 infection but no cART treatment were collected at 4 dpi due to the pick of viral replication with morphological alterations caused by infection to the hCOs. The hCOs with HIV-1 infection and cART treatment showed a stable morphology with limited toxicity. cART treatments were extended to 7 dpi and hCOs were collected. We first analyzed the mRNA expression of HIV receptor and co-receptors in hCOs infected with HIV, either treated with cART or untreated. Interestingly, while there was no significant alteration in CD4 expression in HIV-1 + infected and cART treated hCOs compared to uninfected controls, CCR5 mRNA levels were significantly increased in HIV + and HIV + ART + hCOs (Fig. 4B). More interestingly, HIV-1 infection caused a more significant increase in CXCR4 levels, but its levels were comparable to uninfected controls in hCOs infected with HIV-1 and treated with cART regimen. Next, p24 levels were analyzed in culture media to determine the effectiveness of cART treatments (Fig. 4C). At day 1, low levels of p24 were still detectable in the culture media despite the extensive washing of the viral inoculums. The levels of p24 were increased in both HIV + and HIV + ART + hCOs at 4 dpi with no significant difference between the two groups. Gag p24 average concentration in supernatant was ~ 2700 ng/ml for HIV + and ~ 2500 ng/ml HIV + ART+, with values ranging from 1200 ng/ml to 3700 ng/ml for HIV + and from 1400 ng/ml to 4600 ng/ml for HIV + ART+. At day 7, however, values of p24 of HIV + ART + organoids dropped significantly when compared to HIV + ART + p24 levels at day 4, with an average concentration of 470 ng/ml, and values ranging from 210 ng/ml to 805 ng/ml, suggesting the efficacy of cART regimens in reducing HIV-1 replication and virion production. To further characterize the effect of cART treatment on organoids, gDNA samples from HIV + hCOs and HIV + ART + hCOs were analyzed by ddPCR for the detection of proviral DNA, using primers and probe sets targeting HIV-1 Ψ (psi) packaging element. Consistent with p24 levels in culture media, HIV-1 proviral DNA was significantly reduced in hCOs treated with cART regimens compared to the untreated hCOs (Fig. 4D and E). Lastly, viral RNA transcripts in HIV + and HIV + ART + organoids were also analyzed using quantitative real time RT-qPCR and RNAscope. Consistent with p24 levels and proviral DNA analysis, levels of HIV-1 transcripts Ψ, Gag and Pol were reduced in HIV + ART + when compared to HIV + hCOs (Fig. 4F-H). RNAscope immunostaining of viral RNA transcripts further confirmed the successful suppression of HIV-1 infection by cART treatment in hCOs (Fig. 4I). To gain more insight into cytotoxicity induced by HIV-1 infection and cART regimens, uninfected control, HIV infected and HIV infected and treated with cART regimen hCOs, were processed by immunostaining for cleaved caspase-3, an apoptosis marker (Fig. 4J). HIV-1 infection increased the expression of cleaved caspase-3 when compared to the uninfected controls with limited additional effect caused by cART treatment.

## Discussion

Cerebral organoids are three-dimensional structures that resemble the organization and cell types found in the human brain. They are derived from human pluripotent stem cells and contain different brain cell types, including neurons, astrocytes, and other supporting cells (Lancaster et al. 2013). In the last ten years, COs have been extensively used for a variety of purposes in scientific research. They have been used to simulate various neurological disorders, including microcephaly, Alzheimer’s disease, and different types of neurodegenerative disorders. Additionally, they have been employed to model neurodevelopmental diseases, such as Timothy syndrome, Angelman syndrome, and tuberous sclerosis (Eichmüller and Knoblich 2022). Further, cerebral organoids have been used to assess the effects of drugs and compounds on the human brain (Fan et al. 2022). Recently, cerebral organoids have emerged as a significant tool for investigating neurotropic viruses, in particular HIV (Swingler et al. 2023), even with limited studies using human brain organoids as a model for HIV infection have been reported (dos Reis et al. 2020; Gumbs et al. 2022). The first model of 3D human brain organoids (hBORG) with integrated microglia (MG) was developed by dos Reis et al. (dos Reis et al. 2020; Dos Reis et al. 2023). hBORGs were derived from neuronal progenitor cells (NPCs), thus they consisted solely of neurons and astrocytes. The microglia (MG) were infected with HIV before being integrated into the hBORGs. The model proposed by dos Reis et al. offered progress in modeling HIV infection and capturing certain aspects of HIV-related CNS disease. However, the inclusion of exogenously introduced microglial cells in the organoids may not necessarily represent the microglial structure seen in in vivo conditions. Incorporating microglia presents the greatest difficulty when constructing an organoid model for studying HIV infection. Indeed, the importance of microglia stems from their essential role, alongside macrophages, as major cell types accountable for HIV infection and replication within the brain (Wiley et al. 1999). More recently, Gumbs et al. described a model of microglia-containing cerebral organoids, which they successfully infected with HIV (Gumbs et al. 2022). The model was derived from iPSCs and showed presence of cells from the three germ layers at initial stages of organoid generation (Ormel et al. 2018). In our study, by using similar approach with few alterations and modifications in generation of hCOs, we were able to recapitulate the microglial incorporation into hCOs and their susceptibility to HIV-1 infection. Moreover, for the first time, we were able to propose that cerebral organoids might be a superior tool for studying the effects of cART and neuropathogenesis of HIV-1. This hCO model represents a distinctive and innovative platform that can aide in advancing in vitro research on neuroHIV.

Studying neuroHIV has been challenging due to the limitations of the in vitro models. To capture the dynamic cell-to-cell interactions observed in the intact brain, it is essential to utilize a primary culture system that incorporates both neurons and glial cells, thus mimicking the cellular composition typically found in the brain. In vitro models usually lack the complexity of the in vivo environment, including the intricate interactions between different cell types, the blood-brain barrier, and the immune system. These interactions are particularly relevant when studying neuropathology of HIV infection, where infected microglial cells can exert neurotoxic effects on neurons that are not susceptible to HIV infection (Kovalevich and Langford 2012). NeuroHIV 2D systems usually comprise of co-culture models of various cell types relevant to the CNS, such as neurons, astrocytes, and microglia. However, since in vitro models typically focus on specific cell types, such as neurons and glial cells, they neglect the contributions of other cell types and interactions among them that may be relevant in HAND pathogenesis. A significant limitation of many existing protocols in generating hCOs is their exclusive emphasis on neurons and astrocytes, without the inclusion of microglia (Lancaster and Knoblich 2014; Pașca 2018). Our model has shown to contain not only astrocytes and neuronal cells, but also microglia, rendering it a valuable tool for investigating the intricate interactions among these cell types and for effectively modeling HIV infection within the brain. Our data have further demonstrated that the hCOs models can support HIV infection and replication. Moreover, the effectiveness of cART in suppressing HIV replication was also established, suggesting that hCOs might serve as a tool to better understanding the efficacy of antiretroviral drugs in inhibiting viral replication, to gain insight in reducing viral reservoirs and mitigating HIV-associated neurotoxicity.

HIV utilizes specific receptors and coreceptors on target cells for the infection. The primary receptor used is CD4, which is a glycoprotein present on the surface of immune cells such as T cells, macrophages, and dendritic cells. In addition to CD4, HIV requires a coreceptor to facilitate fusion and entry into the host cells. The most common coreceptors used by HIV are chemokine receptors known as CCR5 and CXCR4 (Wilen et al. 2012). There are limited studies on alterations in expression of HIV receptor and co-receptors following by HIV infection. A cross-sectional study performed on PWH have shown down-regulation of CXCR4 expression on CD4 + and CD8 + T cells, as well as CD14 + monocytes. However, expression of CCR5 on CD4 + T cells was found to be up regulated when compared to uninfected controls (Ostrowski et al. 1998). Other studies have proposed a positive correlation between density of CCR5 and HIV cells infectability, although the main goal was improving the HIV infection (Platt et al. 1998; Reynes et al. 2000). In order to gain more insight on CD4, CCR5 and CXCR4 expressions following HIV infection, we performed a quantitative analysis of these molecules in hCOs. Interestingly, expression of HIV co-receptors CCR5 and CXCR4 is significantly upregulated following HIV infection. More interestingly, CXCR4 expression is shown to be significantly reduced after cART treatment. CCR5 expression level has been studied in correlation with cognitive deficits in PWH, since patients treated with maraviroc, a CCR5 antagonist, have shown improvement in neurocognitive function (Ndhlovu et al. 2014; Gates et al. 2016; Barber et al. 2018). HIV animal models subjected to *Ccr5* knockout or knockdown have shown to be able to improve from cognitive deficits, following reduction in microgliosis and neuroinflammation (Riviere-Cazaux et al. 2022). Further investigation of the roles of CCR5 and CXCR4 in hCOs may reveal novel and functional roles in HIV neuropathogenesis.

Although recent improvements in establishing protocols for generating cerebral organoids have allowed us to generate 3D models that can closely model the human brain in vitro, some limitations are still present. One of the major limitations is the lack of vascularization, that would improve nutrients diffusion into the hCOs, and would allow for the formation of BBB models (Miller et al. 2012). The permeability of the BBB is precisely regulated, but HIV infection and the presence of viral proteins have been demonstrated to disrupt these regulatory processes, thereby contributing to the development of HIV-1-associated neuropathogenesis (Strazza et al. 2011). The hCOs used in our study lack the brain microvascular endothelial cells (BMEC), a central element in the formation of BBB. Some recent studies have proposed co-culture systems with endothelial cells or assembloids between hCOs and vascular organoids (Pham et al. 2018; Sun et al. 2022), but the protocols do not offer yet a reliable approach to generate vascularized cerebral organoids. Another limitation in the hCO models is the lack of a functional immune system. This restricts the ability of hCOs to fully replicate the complex interactions between viral infections, immune responses, and the brain microenvironment seen in vivo. This is particularly important in studying HIV, since the resting memory CD4 + T cells are an important viral reservoir (Eisele and Siliciano 2012). Viral rebound in the periphery can contribute to the development or exacerbation of HAND. The proinflammatory mediators, as well as viral proteins, released by infected cells in the peripheral circulation might permeate into the CNS, leading to an exacerbation of inflammatory responses within the CNS (Sonti et al. 2021). Additional studies are needed to further improve cerebral organoids, but their innovative approach represents a significant improvement in the field, enabling the possibility of more accurate and comprehensive studies of HIV infections in the context of the human brain.

Despite the limitations discussed, our results suggest that hCOs might be an innovative model system with enormous potential for investigating the consequences of HIV infection on the CNS, advancing our comprehension of the neuropathogenesis associated with HIV-associated neurocognitive disorders and HIV latency in the brain, and advancing the assessment of novel therapeutic and curative approaches.

## Materials and methods

### Cell culture

HDFa were cultured in FibroLife Basal Medium (LIFELINE Cell Technology cat.no LL-0011) supplemented with rh FGF basic (5 ng/mL), rh insulin (5 µg/mL), Ascorbic Acid (50 µg/mL), L-Glutamine (7.5 mM), hydrocortisone hemi-succinate (1 µg/mL), FBS 2%. Gentamicin (30 µg/mL) and Amphotericin B (15 ng/mL). All these supplements were provided with the FibroLife S2 LifeFactors Kit (LIFELINE Cell Technology cat.no LL-0011). The HDFa supplemented medium was changed every other day and the cells culture was incubated at 37 °C providing with 5% CO2.

### Generation of hiPSCs

HDFa from different donors (LIFELINE Cell Technology cat.no FC-0001) were used to generate hiPSC. HDFa were reprogrammed to hiPSCs with ReproRNA Kit (Stem Cell Technologies cat.no 05930) following the manufacturer’s instructions. Briefly, hiPSC HDFa were plate in a 6-wells plate, previously coated with Matrigel® (Corning® cat.no 354,277), at the confluency of 100,000 HDFa per wells. The day after the HDFa were transfected with the ReproRNA™-OKSGM, a non-integrating, self-replicating RNA-based reprogramming vector which expresses Oct-3/4, Klf-4, Sox2, Glis1, c-Myc, and a puromycin-resistant cassette. The so transfected HDFa were cultured in a selective growth medium made of Advanced DMEM (Thermo Fisher cat.no 12,491,015) supplemented with 10% FBS, 200 mM L-Glutamine (STEMCELL Technology cat.no 07100), 0.5 mg/mL Recombinant B18R Protein at the final concentration of 175 ng/mL (STEMCELL Technology cat.no 78,075) and in presence of 1 mg/mL solution of puromycin (STEMCELL Technology cat.no 73,342) at the final concentration 0.8 µg/mL. The selective growth medium was changed every day for 1 week and then it was replaced by complete ReproTeSR™ medium (STEMCELL Technology cat.no 05926) without puromycin and supplemented with 0.5 mg/mL Recombinant B18R Protein at the final concentration of 175 ng/mL for an additional week. Two weeks after transfection, ReproTeSR™ medium without B18R was daily change until hiPSC colonies formed and were ready to be manually isolated and cut into small fragments with a 22-gauge needle. Then the colony fragments were scraped and aspirated using a 200 µL pipettor with a filtered tip. The hiPSC colony fragments were plated on culture-ware coated with Matrigel® and containing hiPSC maintenance mTeSR™ Plus complete medium (STEMCELL Technology cat.no 100–0276) supplemented with Y-27,632 (STEMCELL Technology cat.no 72,302) at a final concentration of 10 µM. After 24 h, the maintenance medium was replaced to remove the Y-27,632 and the hiPSC were incubated at 37 °C providing with 5% CO2. The hiPSC maintenance medium was changed every other day until the hiPSC colonies reach the confluency of 70%.

### Generation of human cerebral organoids (hCOs)

Newly generated hiPSCs (Fig. 1) were used for creating hCOs by using STEMdiff™ Cerebral Organoid Kit (STEMCELL, 08570), and following manufacturer’s instructions with few modifications. Culture media were freshly prepared for each stage of embryoid body (EB) formation, induction, expansion, and maturation and used at room temperature (RT). On day 0, hiPSCs were detached using the Gentle Cell Dissociation Reagent (STEMCELL, 100–0485) at 37˚C and cells were resuspended in mTeSR™ Plus media (STEMCELL, 100–0276). After counting, cell mixture was centrifuged at 300 g for 5 min before EB seeding. 12,000 hiPSCs were used per EBs. The cell pellet was resuspended in EB seeding medium containing Y-27,632 (STEMCELL, 72,302) at a final concentration of 10 µM. 100 µL of cell suspension was seeded in each well of 96-Well Ultra-Low Attachment Round-Bottom plate (Millipore Sigma, CLS7007). The cells were kept in incubator at 37˚C with 5% CO_2_. On day 2 and day 4, 100 µL of EB Formation Medium was added per well. On day 6, each EB was transferred into a single well of 24-Well Ultra-Low Attachment plates (Millipore Sigma, CLS3473) containing Induction Medium. They were incubated at 37˚C under 5% CO_2_ condition for 48 h. On day 8, each EB was embedded in 15 µL of Matrigel® (CORNING, 354,277) on the organoid embedding sheets (STEMCELL, 08579). The droplets of embedded EBs were transferred into each well of 6-Well Ultra-Low Adherent plates containing Expansion Medium. A maximum of 16 EBs were transferred into each well. EBs were then incubated at 37˚C under 5% CO_2_ condition until the EBs exhibit multi-budding structures. On day 12, a full-mediachange took place with the Maturation Medium. EBs were then placed on an orbital shaker and incubated at 37˚C with 5% CO_2_. A half-media change was performed every 3 to 4 days during and after maturation with identical conditions.

### HIV infection of hCOs and cART treatments

To characterize HIV-1 infection in cerebral organoids, 28 hCOs were incubated with 300 ng NL4.3 HIV-1 EGFP BaL reporter virus. First, hCOs were seeded in 96-well-plates and inoculated with 100 µl in OptiMEM containing diluted virus and *Polybrene* at a final concentration of 5 µg/ml. hCOs were subjected to spinoculation at 1200 *g* for 2 h at 32 °C, then placed in incubators at 37 °C overnight. The following day, inoculum was removed, hCOs were washed twice in PBS, plated in ultra-low attachment 24 well-plates, and incubated at 37 °C in complete hCO media on a continuous shaker. Fourteen of hCOs were left untreated while fourteen hCOs were treated with a cART regimen (Raltegravir-10 µg/ml, Tenofovir disoproxil- 2 µg/ml, Emtricitabine-2 µg/ml). cART treatments and half media changes were performed daily during the duration of the experiments. HIV-infected hCOs were collected at day 4 based on the cytopathic effects observed. HIV-infected and cART-treated hCOs were collected at day 7. Supernatants were collected daily, and Gag p24 ELISA was performed using supernatants at day 1, 4 and day 7. For gDNA analysis, four individual hCOs were used per each condition. For immunofluorescence, four individual hCOs were used per condition. For RNA analysis, six hCOs per condition were used. Fourteen hCOs were left uninfected and used as controls.

### Immunocytochemistry (ICC)

250,000 hiPSC were plated in 2-well chamber slides previously coated with Matrigel®. The cells were washed with D-PBS (without Ca + + and Mg++) and fixed with cold 4% PFA in PBS solution for 10 min at RT, permeabilized with 0.25% Tx-100 in PBS for 5 min at RT and blocked with 5% Normal Donkey Serum (NDS) in 0.1% Bovine Serum Albumin in PBS (BSA/PBS). The primary antibody was diluted in 0.1% BSA/PBS individually and incubated at 4˚C overnight with gentle rocking. Primary antibodies used are listed in Table 1. The corresponding secondary antibodies of Alexa Fluor 488 (Donkey Anti-Mouse), Alexa Fluor 488 (Donkey Anti-Rabbit), Alexa Fluor 594 (Donkey Anti-Mouse), and Alexa Fluor 594 (Donkey Anti-Rabbit) were diluted in 0.1% BSA/PBS solution at dilution 1:400 and incubated for 2 h in the dark container at RT with gentle rocking. The chambers were disassembled from the slides before cover-slipping with DAPI mounting medium. The coverslips were secured with clear nail polish. The slides were imaged with a Keyence microscope after 30 min.

**Table 1:** List of primary antibodies used

Primary Antibody	Cell type	Reference	Vendor	Cat #
Anti-Human OCT4 (OCT3), Clone 3A2A20	iPSCs	(Huangfu et al. 2008; Medvedev et al. 2010; Marei et al. 2017; Sagar et al. 2023)	STEMCELL Technologies	60,093
Anti-Human SSEA-4 Antibody, Clone MC-813-70	iPSCs	(Huangfu et al. 2008; Marchetto et al. 2009; Medvedev et al. 2010; Marei et al. 2017)	STEMCELL Technologies	60,062
Anti-Human TRA-1-60, Clone TRA-1-60R	iPSCs	(Huangfu et al. 2008; Marchetto et al. 2009; Medvedev et al. 2010; Marei et al. 2017; Sagar et al. 2023)	STEMCELL Technologies	60,064
Anti-Olig2	Oligodendrocytes	(Yokoo et al. 2004)	Abcam	ab254043
Anti-GFAP (GA5)	Astrocytes	(Wu et al. 2015; Sami Saribas et al. 2017)	MilliporeSigma	MAB360
Anti-Iba1	Microglia	(Hovens et al. 2014; Hendrickx et al. 2017; González Ibanez et al. 2019)	FUJIFILM Wako	019-19741
Anti-MAP2 (D5G1)	Neuronal cells	(Sami Saribas et al. 2017; Donadoni et al. 2019; Sagar et al. 2023)	Cell Signaling Technology	8707 S
Anti-Tubulin Beta 3 (TUJ-1)	Neuronal cells	(Sami Saribas et al. 2017; Das et al. 2020; Sagar et al. 2023)	Santa Cruz Biotechnology	sc-58,888
Anti-Ki67	N/A	N/A	Abcam	ab15580
Anti-Pax6	Neural stem cells	(Bell et al. 2019)	Biolegend	901,301
Anti-SOX1	Neural stem cells	(Bell et al. 2019)	R&D Systems	AF3369
Anti-TMEM119	Microglia	(González Ibanez et al. 2019)	Abcam	ab185333
Anti-GFP (Living Colors®)	N/A	N/A	Takata Bio	632,375
anti-HIV-1 NEF Protein (EH1)	N/A	(Sami Saribas et al. 2017; Yarandi et al. 2020)	NIH HIV Reagent Program	ARP-3689

### RT-PCR analysis of pluripotency and differentiation markers

Total RNA was extracted from HDFa, iPSCs and hCOs using an RNA extraction kit (New England Biolabs) according to the manufacturer’s instructions. RT–PCR reactions were performed as previously described (Donadoni et al. 2019). Briefly, cDNAs were synthesized by using M-MLV Reverse Transcriptase (Invitrogen) followed by removal of RNA templates by RNase H digestion. A total of 50 ng cDNA was used as template for PCR reactions. Pluripotency markers OCT4 and SOX2, and differentiation markers, such as astrocytic structural marker, GFAP, neuronal differentiation markers, TUJ1 and MAP2, and oligodendroglia marker, Olig2, were amplified by PCR. GAPDH mRNA was amplified from the same set as internal controls. List of primers used is available in Table 2. Amplified products were resolved on 1% DNA agarose gels and visualized by ethidium bromide staining.

**Table 2:** List of primers and probes used

Gene	Primer name	Sequence (5’→3’)	Reference
OCT4	Fw	GGAAGGAATTGGGAACACAAAGG	(Marsoner et al. 2016)
	Rv	AACTTCACCTTCCCTCCAACCA	
SOX2	Fw	GGATAAGTACACGCTGCCC	(Xu et al. 2018)
	Rv	ATGTGCGCGTAACTGTCCAT	
GFAP	Fw	GGATCTGGAGAGGAAGATTG	N/A
	Rv	CGCCATTGCCTCATACT	
Iba-1	Fw	CAAGACTCACCTAGAGCTAAA	N/A
	Rv	CAGGGCAACTCAGAGATAG	
TUJ1	Fw	CTCAGGGGCCTTTGGACATC	(Jouhilahti et al. 2008)
	Rv	CAGGCAGTCGCAGTTTTCAC	
MAP2	Fw	GACCCTTAGCAGGAGTTTAG	N/A
	Rv	GGTGGCTGGAAGGTAATC	
Olig2	Fw	CAGCTGCGTCTCAAGAT	N/A
	Rv	CAGTCGCTTCATCTCCTC	
GAPDH	Fw	ACCACAGTCCATGCCATCAC	(Donadoni et al. 2022)
	Rv	TCCACCACCCTGTTGCTGTA	
CD4	Fw	TTCAGGACACAGGGAAATCAGGGTT	N/A
	Rv	GGAAGTGGTGAGGAAGGGTAGGAAG	
CCR5	Fw	TCTCTTCTGGGCTCCCTACAACATT	N/A
	Rv	TCTCTGTCACCTGCATAGCTTGGTC	
CXCR4	Fw	CTTTGTCATCACGCTTCCCTTCTGG	N/A
	Rv	AGGACACTGCTGTAGAGGTTGACTG	
β–Actin	Fw	GCATCCTCACCCTGAAGTA	N/A
	Rv	CACGCAGCTCATTGTAGAAG	
	Probe-HEX	ACCAACTGGGACGACATGGAGAAA	
Ψ PSI	Fw	CAGGACTCGGCTTGCTGAAG	(Bruner et al. 2019)
	Rv	GCACCCATCTCTCTCCTTCTAGC	
	Probe-FAM	TTTTGGCGTACTCACCAGT	
HsTERT	Fw	TGGAGCAAGTTGCAAAGCAT	(Liu et al. 2023)
	Rv	CAGAGCCTTGCACAGAATCC	
	Probe-HEX	CCGGCCTCAGCATGCGCCTG	
GagD	Fw	AAGTAGTGTGTGCCCGTCTG	N/A
	Rv	TCGAGAGATCTCCTCTGGCT	
	Probe-FAM	CTGTTCGGGCGCCACTGCTA	
Pol	Fw	ACAGACAATGGCAGCAATTTCACC	N/A
	Rv	TGCCAAATTCCTGCTTGATCCC	
	Probe-FAM	CGCCCACCAACAGGCGGCCTTAACTG	

### hiPSC genetic analysis

The hiPSCs genetic analysis was performed to detect karyotypic abnormalities using hPSC Genetic Analysis Kit (STEMCELL Technology), following manufacturer’s instruction. Briefly, 300 ng of gDNA was analyzed using a qPCR-based technology to calculate copies number of chromosomes 1q, 4p, 8q, 10p,12p,17q,18q, 20q and Xp, in a LightCycler 96 instrument (Roche, Indianapolis, IN, USA). Results obtained were analyzing using application provided by the company (www.stemcell.com/geneticanalysisapp).

### Real-time RT-qPCR

RT-qPCR assays for HIV receptors CD4, CCR5 and CXCR were performed using RNA from hiPSCs from three different donors and eight different hCOs, with hCOs combined in two. RT-qPCR assays for characterization of HIV receptors following HIV infection were performed using RNA from uninfected and infected hCOs. RNA was extracted using an RNA extraction kit (New England Biolabs) according to the manufacturer’s instructions. RT-qPCR was performed using Luna® Universal One-Step RT-qPCR Kit (New England Biolabs) in a LightCycler 96 instrument (Roche, Indianapolis, IN, USA). The reaction mixtures contained: 1 × Luna Universal One-Step Reaction Mix, 1 × Luna WarmStart® RT Enzyme Mix, 0.4 µM of forward primer, 0.4 µM of reverse primer, 100 ng of template RNA, and nuclease-free water to a final volume of 20 µL. The protocol was reverse transcription at 55 °C for 10 min, initial denaturation at 95 °C for 60 s, followed by 45 cycles of denaturation at 95 °C for 10 s, and extensions at 60 °C for 30 s, with single acquisition. The amplification steps were then followed by melting steps: initial denaturation at 95 °C for 10 s, followed by 60 s at 65 °C, and temperature increase with continuous readings for 1 s to reach 97 °C. RT-qPCR assays for HIV infection characterization were performed on uninfected or infected hCOs, with or without cART treatments. RNA was extracted using an RNA extraction kit (New England Biolabs) according to the manufacturer’s instructions. RT-qPCR was performed using Luna® Universal Probe One-Step RT-qPCR Kit (New England Biolabs) in a LightCycler 96 instrument (Roche, Indianapolis, IN, USA). The reaction mixtures contained: 1 × Luna Universal Probe One-Step Reaction Mix, 1 × Luna WarmStart® RT Enzyme Mix, 0.4 µM of forward primer, 0.4 µM of reverse primer, 0.2 µM of probe, 25 ng of template RNA, and nuclease-free water to a final volume of 20 µL. The protocol was reverse transcription at 55 °C for 10 min, initial denaturation at 95 °C for 60 s, followed by 45 cycles of denaturation at 95 °C for 10 s, and extensions at 60 °C for 30 s, with single acquisition. The primers used in all the RT-qPCR assay are listed in Table 2.

### NL4.3 HIV-1 EGFP BaL viral preparation

NL4.3 HIV-1 EGFP BaL (M-tropic HIV-1 strain) was prepared transfecting the pNL4.3-EGFP-BaL plasmid (containing the HIV-1 NL4.3 strain with the env of BaL strain and the gene encoding EGFP between env and nef without affecting expression of any HIV gene) in HEK 293 T cells. Briefly, 10ug of pNL4.3-EGFP-BaL plasmid were transfected in HEK 293 T cells at the confluency of 60% in 100 mm dish, using Lipofectamine™ 3000 Transfection Reagent (InvitrogenTM cat.no L3000015). After 24 h the media with DNA-lipid complex was replaced with DMEM 5% FBS. After 48 h and 72 h the NL4.3 HIV-1 EGFP BaL virus was collected and concentrated according to the pseudotyped HIV-1-based lentiviral vector (Kutner et al. 2009). Briefly, the collected cell supernatant was centrifuged at 500 g for 10 min at 25 °C to remove cells and large cell debris. Then the pooled supernatants were filtered using a 0.45 μm PES filter (Corning, cat.no. 430,768). Until 32mL aliquots of filtered lentivirus containing cell culture supernatant were transferred into each of the 6 Ultra-clear SW28 tubes (Beckman, cat.no 344,058) and 4 mL of 20% sucrose solution (20 g of UltraPure sucrose, 100mM NaCl, 20mM HEPES pH 7.4 and 1mM EDTA) was released all the way to the bottom of the SW28 tube filled with the filtered lentivirus-containing supernatant. The SW28 tubes were centrifuged for 2 h at 82,700 g and 4 °C using an ultracentrifuge. After centrifugation the supernatant was poured off and the pellet at the bottom of the tube was resuspended in 100 µL of PBS without Ca/Mg per tube. The tubes were incubated at 4 °C for 2 h on a shaking platform and then they were spined at 500 g for 1 min at 25 °C to collect the lentivirus-containing liquids. Finally, the lentivirus was aliquoted in screwcap cryo-vials in 30 µL portions, snap-frozen in crushed dry ice and stored at -80 °C.

### HIV-1 gag p24 ELISA (enzyme-Linked immunosorbent assay)

NL4.3 HIV-1 EGFP BaL titer was measured by p24 Gag ELISA (Advanced BioScience Laboratories, Inc.), following instructions provided by the manufacturer. After HIV infection and ART treatment of hCOs, supernatants were collected, and levels of HIV-1 viral load were also quantified by p24 Gag ELISA. Proper dilutions of supernatant were made to be within the range of the assay.

### Droplet Digital Polymerase Chain Reaction (ddPCR)

To analyze HIV-1 DNA in hCOs, ddPCR amplifying HIV-1 Ψ gene and human TERT as a reference gene was performed, using the QX200™ Droplet Digital™ PCR System (Bio-Rad, Hercules, CA, USA). The ddPCR reaction mixtures was prepared adding the following reagents: 1 × ddPCR™ Supermix for Probes (No dUTP) (Bio-Rad, Pleasanton, CA, USA), 500 nM of HIV-1 Ψ forward primer, 500 nM HIV-1 Ψ reverse primer, 500 nM of HIV-1 Ψ FAM probe, 500 nM of HsTERT forward primer, 500 nM HsTERT reverse primer, 500 nM of HsTERT HEX probe, 50 ng gDNA and water to a final volume of 22 µL. The ddPCR droplet and plate preparation were performed as previously described (Donadoni et al. 2019). The thermocycling protocol was previously described: initial denaturation at 95 °C for 10 min, then 45 cycles of denaturation at 94 °C for 30 s, annealing and extension at 59 °C for 1 min, followed by a final last incubation at 98 °C for 10 min and storage at 4 °C (Bruner et al. 2019). After amplification, positive and negative droplets of each sample were read and analyzed and graphed as HIV-1 Ψ copies per 1 million of cells. List of primers and probes use in Table 2.

### Fixation and embedding of human cerebral organoids (hCOs)

Before immunoanalytical assays, hCOs were collected and washed three times with D-PBSfor 10 min. hCOs were fixed with 4% PFA overnight at 4˚C for 16 h, washed with 0.1% PBS-T, and kept in D-PBS for 1 to 7 days at 4˚C. The 30% Sucrose in D-PBS solution was used for dehydration for 1–2 days until the hCOs were submerged. hCOs were then embedded in Optimal Cutting Temperature Embedding Medium (OCT) to obtain 10 μm thick sections at cryostat and air-dried for 20 min at RT before staining or storing at -80˚C.

### Immunofluorescence

The hCO sections were washed with 0.05% Tween-20 in PBS (PBS-T) to remove OCT, permeabilized for 15 min with 0.3% Tx-100 in PBS and blocked with 5% NDS for 1 h at RT in a humidifying chamber. The primary antibodies were diluted in the Dako Antibody Diluent (Agency for Science, Technology and Research, S3022) and incubated at 4˚C overnight in a humidifying chamber. Primary antibodies used are listed in Table 1. The secondary antibodies of Alexa Fluor 488 (Donkey Anti-Mouse), Alexa Fluor 488 (Donkey Anti-Rabbit), Alexa Fluor 594 (Donkey Anti-Mouse), and Alexa Fluor 594 (Donkey Anti-Rabbit) were diluted in the ratio of 1:1000 and incubated at RT for 1 h in a lightless humidifying chamber. The slides were cover slipped with DAPI mounting medium (Invitrogen, P36935). After 30 min of solidification at RT, the slides were imaged with a Keyence microscope.

### RNAscope

The protocol of the RNAscope® 2.5 HD Detection Reagents-RED assay (ACD™, 322,360) was followed for the frozen sections of hCOs with modifications. The OCT reagent was washed off from cryosections with PBS-T before incubating at 60˚C for 30 min. The slides were fixed with cold 4% PFA in PBS for 15 min at 4˚C, dehydrated with 50%, 70%, 90%, and 100% Ethanol (EtOH), respectively. The sections were incubated with RNAscope® Hydrogen Peroxide for 10 min and rinsed with DI water. For antigen retrieval, the slides were kept in the RNAscope™ Target Retrieval Reagent (ACD, 322,000) for 5 min at 98–102˚C and equilibrated to RT before rinsing. A barrier was created around the sections with a ImmEdge® Pen (Vector Laboratories, H-400), then slides were stored at 4˚C overnight. The sections were blocked for 10 min with Boxall Blocking reagent at RT and treated with RNAscope® Protease Pluss (ACD, 322,330) for 30 min at 40˚C inside a humidifying chamber. The slides were washed with the RNAscope wash buffer between each treatment. Sections were hybridized with HIV probe (ACD, 444,061), Positive probe (ACD, 313,901), and Negative probe (ACD, 310,043) for 2 h at 40˚C in the humidifying chamber. The Amplification 1–6 solutions were applied at their specific conditions. The chromogen development was achieved with the RED B and RED A solution, which had a ratio of 1:60 and incubated for 10 min at RT in the dark. The sections were counter-stained with Hematoxylin QS and dehydrated with the indicated technique above. The slides were cover-slipped with permanent mounting medium (Vector, H-500-60).

#### Immunohistochemistry (IHC)

After RNAscope, the IHC was performed before counter-staining. The sections were blocked with Serum-Free Protein Block (Dako, X090930-2) reagent for 30 min at RT. The primary antibodies were incubated overnight at 4˚C, after diluting with Dako Antibody Diluent. The secondary antibody of Dako EnVision + System-HRP Labeled Polymer anti ‘host’ was applied for 30 min at RT in the dark. The DAB + Chromogen solution (Dako, K3468) was kept for 1–2 min and deactivated with DI water.

#### Statistical analysis

All values presented on the graphs are given a mean ± SEM. Analysis of variance and unpaired Student’s t-test were used to analyze the statistical significance. p-values of < 0.05 were considered statistically significant.

## Data Availability

No datasets were generated or analysed during the current study.
